# The State Lunatic Asylum at Austin

**Published:** 1891-06

**Authors:** 


### EDITORIAL DEPARTMENT.

F. E. DANIEL, M. D., EDITOR AND PUBLISHER.

THIS JOURNAL, by unanimous vote of the members, was made the Official Organ of the Austin District Medical Society.

COLLABORATORS:

*Dr. R. M. Swearingen, Austin.*
*Prof. B. E. Hadra, M. D., Galveston.*
*Prof. Geo. Cupples, M. D., San Antonio.*
*Dr. T. C. Osborn, Cleburne, Texas.*
*Dr E. J. Doering, Chicago.*
*Dr. E. J. Beall, Fort Worth.*
*Dr Odo Betz, Germany.*
*Prof. W. B. Rogers, M. D., Memphis.*
*Dr. J. W. McLaughlin, Austin.*
*Dr. T. J. Tyner, Austin.*
*Prof. J. F. Y. Paine, M. D., Galveston.*
*Dr. R. H. L. Bibb, Mexico.*
*Dr. T. J. Bennett. Austin.*
*Dr. Bat Smith, Wharton.*
*Dr. E. Meierhof, New York.*
*L. H. Luce, M. D., West Tisbury, Mass.*

### THE STATE LUNATIC ASYLUM AT AUSTIN.

During the last administration there was so much complaint of the condition and management of the State Lunatic Asylum at Austin, and there was such a continual uproar and confusion out there, that it is a real pleasure to be able to chronicle so marked a change for the better as has obtained under the new administration. A recent visit and thorough inspection of the institution by a representative of the JOURNAL, impressed us very forcibly with the fact that Superintendent Reeves and his courteous aids, Assistant Physicians Worsham and Maxwell, are "the right men in the right place," and that the honor of Texas and the welfare of the large number of unfortunates are safe in the hands of these efficient officers.

It requires an executive and administrative ability of no mean order, to successfully and harmoniously conduct the manifold and diversified affairs of an institution of such magnitude; and yet we found everything, from the office to the kitchen and laundry, systematized, and moving along smoothly and without friction.

There are in all, male and female, white and colored, 635 patients in the asylum; (of the latter there are 156). Including officers and attendants, this makes up a community of nearly eight hundred souls—quite a village population; and the most conspicuous thing we observed, next to the scrupulous cleanliness of everything, was the absence of noise. People naturally associate howling and hideous cries, revolting sights and more or less confusion with lunatic asylums; and visions of straight jackets and dungeons rise up. But all that is changed. The officers say they seldom, very rarely indeed, have to resort to physical restraint, and find kind and gentle, but firm, management, and the benign influence of beautiful surroundings—flowers, fountains and forest trees, green lawns and innocent sports conducive to mental quiet, and quite sufficient to control the patients. It is even rare to have to administer sedatives.

The long halls and dormitories are clean, cool and well ventilated; in fact, scrupulous hygiene is observed everywhere. Many of the male patients prefer to do light work, and acres upon acres of vegetables, enough to supply the institution, are cultivated by them. This form of exercise and mental occupation is, of course, beneficial in its effect, and conducive to physical and mental health.

Great attention is paid to keeping the grounds in order, and to the cultivation of flowers. Pots of geraniums and other flowers were found in the windows and balconies of every hall and dormitory, and the inmates, especially the females, seemed fond of them, and were cheerful and contented.

The institution is filled at present beyond its capacity, though not uncomfortably crowded. There are sixty patients more than there is really provision for; and there is a large number of applications on file for admission, and as soon as one patient is discharged the management know just where a successor is to be found, and they write for the one whose application is next on file, preference being given to the indigent class. In this way the institution is kept full to overflowing, and it is seldom that a patient, *i. e.*, one of the better class, can obtain admittance. Of the 635 patients now in hand, only about 25 are of the latter class.

Does not this clearly demonstrate the necessity for a private institution in Texas, for the benefit of those who are able to pay?

or for increased accommodation by the State? especially when it is remembered that pending their application for admission here the latter class of insane have to be sent to other States for treatment, and the former are incarcerated in foul county jails? Shame, shame; it is an outrage that a civilized community shudders at; but with the already large provision made for them here, and the erection of a new asylum at San Antonio which is nearing completion, and the 700 capacity asylum at Terrell under the efficient management of Superintendent Preston, what else can be done?* The insane in Texas seem to be on the increase *pari passu* with the great influx of population; and it is impossible to provide for them fast enough. The State of Texas is not niggardly iu this matter, and her people feel a just pride in their great charities; it is an evil which must perforce be borne yet awhile. As much opposed as the average legislator is to "appropriations," the purse strings are freely loosened at the call of distress, and no doubt the next legislature will make even more ample provision for the insane of the State. Dr. Reeves has a $5000 appropriation for a new laundry building, and this will enable him to convert the present laundry into a ward, to which he will transfer the colored females. This will afford some relief.

In this connection, the JOURNAL cannot refrain from the expression of admiration for the very remarkable acumen displayed by Governor Hogg in the selection of his appointees—medical and others; it amounts almost to a spirit of divination. Such a knowledge of human nature as he has manifested in his selection of officers, is indeed rare, and is an emphatic vindication of the judgment displayed by the unprecedented popular majority which called him to the high and responsible office. Apparently he has not make a mistake in a single instance, from the construction of the powerful railroad commission to the humblest office; and no complaint has been made against any of his appointees, so far as we know. This certainly is genius. Few

*A convention of county judges of Texas was recently held, and a petition to the Legislature for relief in this respect, and a protest against this state of affairs in the name of humanity, were drawn up, and will be presented.

men similarly situated could have harmonized so many conflicting elements, disappointed so many office seekers, filled so many and such diversified stations with *precisely the right men*, and so escaped censure or mistake. And had he done nothing else to cement the admiration and devotion of his vast constituency, the intelligence, judgment and discrimination with which the Governor has chosen the heads of departments, would have fixed the seal of his greatness, goodness and wisdom. The friends of the large class of unfortunates especially, the insane, the deaf and dumb, the blind, will "rise up and call him blessed" so long as memory lasts, or the human heart warms toward a generous act or beats responsive to human suffering. The welfare of these people has, beyond doubt, been the paramount consideration with him in the selection of those to whose care they are entrusted.

The remarkable absence of acute sickness at the asylum, forcibly impressed us, the hospital being empty at a season when dysentery and other diseases are elsewhere prevalent. This alone testifies to good and efficient management.

